# Case Report: Navigating the flow-dependent interaction between the left internal mammary artery graft and the left anterior descending artery: when deliberate graft occlusion comes into play

**DOI:** 10.3389/fcvm.2026.1865177

**Published:** 2026-08-04

**Authors:** Andreas Y. Andreou

**Affiliations:** 1 Department of Cardiology, Limassol General Hospital, Limassol, Cyprus; 2 Department of Basic and Clinical Sciences, University of Nicosia Medical School, Nicosia, Cyprus

**Keywords:** competitive flow, coronary artery bypass grafting, graft patency, left internal mammary artery, percutaneous coronary intervention

## Abstract

**Background:**

The left internal mammary artery (LIMA) graft constitutes the optimal conduit for bypassing the left anterior descending (LAD) artery. Despite its overall excellent long-term patency, the LIMA graft may fail, most commonly because of competitive flow. In case of a failed LIMA graft, a percutaneous coronary intervention (PCI) of the LAD artery may be required to ensure better outcomes. However, persistent competitive flow from an anatomically patent but functionally occluded LIMA graft after a successful PCI of the LAD artery may adversely affect clinical outcomes by predisposing to in-stent restenosis (ISR) and stent thrombosis.

**Case summary:**

Herein, we present the case of a patient with unstable angina and a failed LIMA graft exhibiting the “string phenomenon” because of competitive flow, necessitating a PCI of a high-grade stenosis in the LAD artery. Reverse LIMA graft flow, which is an indicator of severe competitive flow, was observed immediately after the PCI and during follow-up angiography, which also revealed an ISR of the LAD artery. Stent failure was attributed to the persisting competitive flow and was managed with drug-coated balloon angioplasty. In order to nullify any future deleterious effects of the competitive flow, we applied the “condom technique” to sacrifice the dysfunctional LIMA graft. The first attempt to apply this technique was complicated by the dislodgment of the remnant balloon from the assembly and migration into the distal LAD artery; however, the second attempt was successful in achieving graft occlusion. The patient was discharged home in good condition and remained free of angina or any cardiovascular events at a 6-month follow-up. An angiogram performed seven months after the drug-coated balloon angioplasty for ISR and occlusion of the LIMA graft showed preserved patency of the LAD artery.

**Conclusion:**

Deliberate occlusion of a dysfunctional LIMA graft following a PCI of the LAD artery may help reduce the risk of future episodes of stent failure. Choosing to sacrifice the graft using the “condom technique” is feasible, safe, and effective, provided all steps of the technique are thoroughly reviewed and optimally performed.

## Introduction

Grafting the left internal mammary artery (LIMA) to the left anterior descending (LAD) artery has been the “gold standard” of coronary artery bypass graft surgery (CABG), associated with a significant survival benefit compared with grafting using saphenous vein grafts (SVGs), by virtue of superior long-term patency rates and resistance to atherosclerosis (1). However, LIMA grafts can fail with an incidence rate of 10% over the long term, with competitive flow identified as the most important contributing factor (2–6). In such cases, the LIMA graft usually develops diffuse narrowing (the “string phenomenon”), representing a potentially reversible response to a low-flow state induced by competitive flow (2–9). In case of a failed LIMA graft to the LAD artery, revascularization of the native coronary artery by percutaneous coronary intervention (PCI) may be necessary to achieve better outcomes. However, persistent competitive flow from an anatomically patent but functionally occluded LIMA graft after a successful PCI of the LAD artery may adversely affect clinical outcomes by predisposing to in-stent restenosis (ISR) and stent thrombosis (10–12).

This report presents the case of a patient with a LIMA graft exhibiting the “string phenomenon,” necessitating a PCI for high-grade stenosis in the LAD artery. Reverse LIMA graft flow was observed immediately after the PCI and persisted during follow-up angiography, which also showed ISR. Because the ISR was ascribed to the persisting competitive flow, we proceeded to consider occlusion of the dysfunctional LIMA graft using the “condom technique.” In this study, we discuss the rationale behind our decision to sacrifice the LIMA graft and the technical aspects and pitfalls of the “condom technique,” which, to the best of our knowledge, has not been applied previously for this indication.

## Case presentation

**Figure 1:**
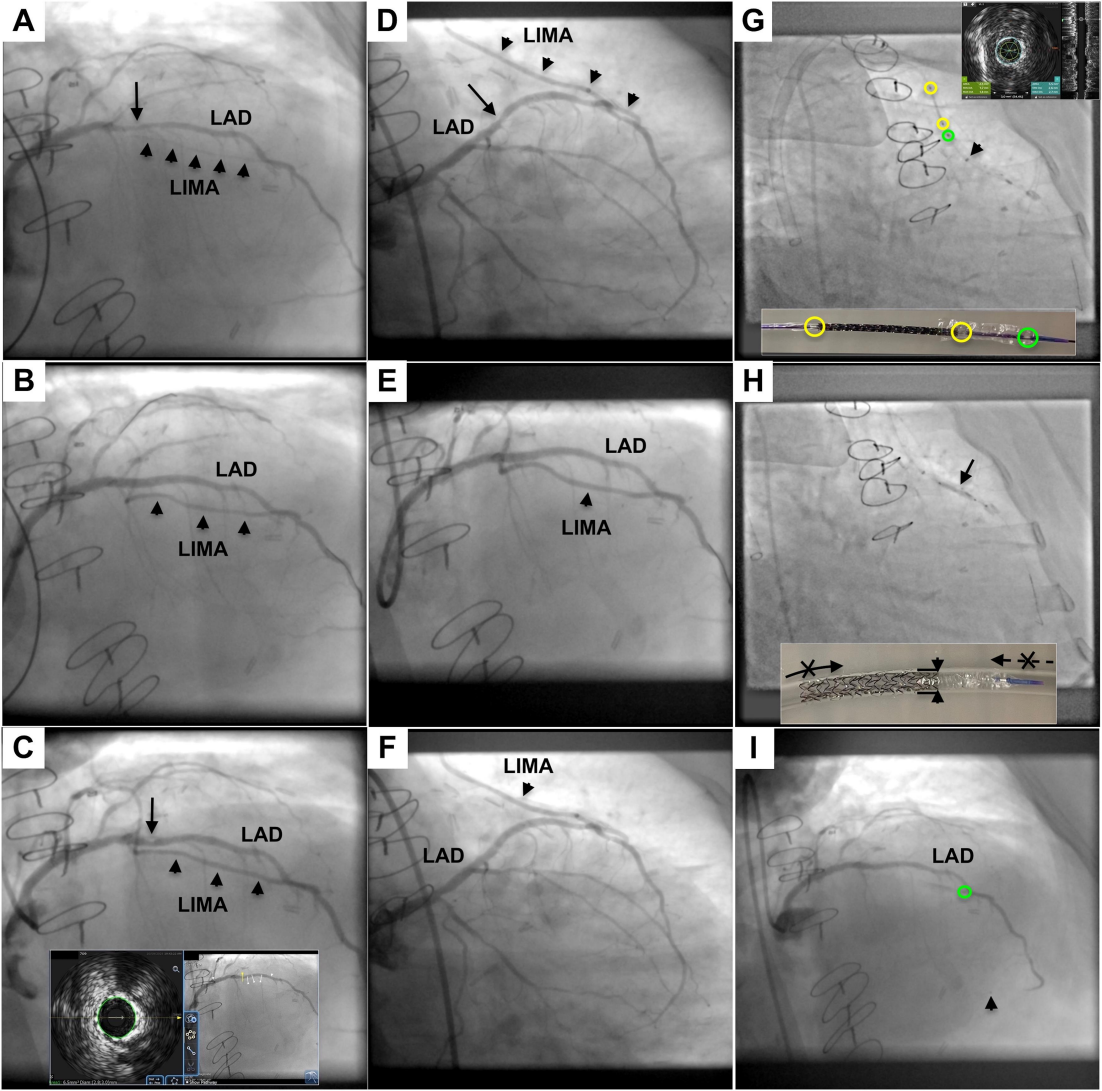
Deliberate occlusion of a dysfunctional LIMA graft using the “condom technique,” conventional angiographic images: **(A)** high-grade stenosis (arrow) of a wrap-around left anterior descending (LAD) artery. The “string phenomenon” (arrowheads) of the left internal mammary artery (LIMA) graft is also shown. **(B)** Good results after drug-eluting stent-assisted angioplasty of the LAD artery. Note the reverse flow through the dilated LIMA graft (arrowheads). **(C)** In-stent restenosis (ISR; arrow) of the LAD artery at the site of a well-expanded stent, showing an intravascular ultrasound (IVUS)-derived minimal stent area of 6.5 mm^2^ (embedded panel). Reverse LIMA graft flow (arrowheads) is also shown. **(D)** ISR (arrow) of the LAD artery and reverse LIMA graft flow (arrowheads) shown in the right anterior oblique (RAO) caudal view. **(E)** and **(F)** Good results after treatment of the ISR by drug-coated balloon angioplasty and reverse LIMA graft flow (arrowhead) depicted in RAO cranial and RAO caudal views, respectively. **(G)** Delivery of the assembly of a cut remnant of the distal part of a 2.25 mm angioplasty balloon mounted on a 2.25 mm stent (embedded bottom panel; the assembly is constructed by advancing the stent as distally as possible inside the balloon remnant, and then the balloon is brought back over the stent) in the LIMA graft through a guideliner catheter (arrowhead) to perform the “condom technique.” The length of the stent is indicated by the two dots of markers seen on both ends of the stent delivery balloon (yellow circles), while the distal end of the remnant of the angioplasty balloon is indicated by the green circle. Note that the dysfunctional LIMA graft was atheromatous (embedded IVUS image). **(H)** Stent deployment (arrow) proximal to the distal LIMA graft anastomosis, fixing the proximal part of the balloon remnant (embedded bottom panel; black lines) on the LIMA graft wall. This way, a dead end is created at the stent–balloon interface (arrowheads), preventing retrograde blood flow from the native coronary artery to the corresponding graft (dashed arrow). Also, the fixed balloon remnant hanging in the graft lumen represents another dead end preventing blood flow from the graft to the corresponding native coronary artery (solid arrow). **(I)** Absence of a retrograde flow from the LAD artery to the LIMA graft, indicating successful occlusion of the graft. The distal end of the remnant of the angioplasty balloon, whose proximal part became trapped between the stent and graft wall, thereby forming a dead end, is indicated by the dot of the marker marked with a green circle. The dot of the marker marked with an arrowhead represents the distal end of the remnant balloon, which dislodged from the assembly during the first attempt to perform the “condom technique”; it was deliberately wedged in the distal part of the wrap-around LAD artery, causing occlusion.

**Figure 2:**
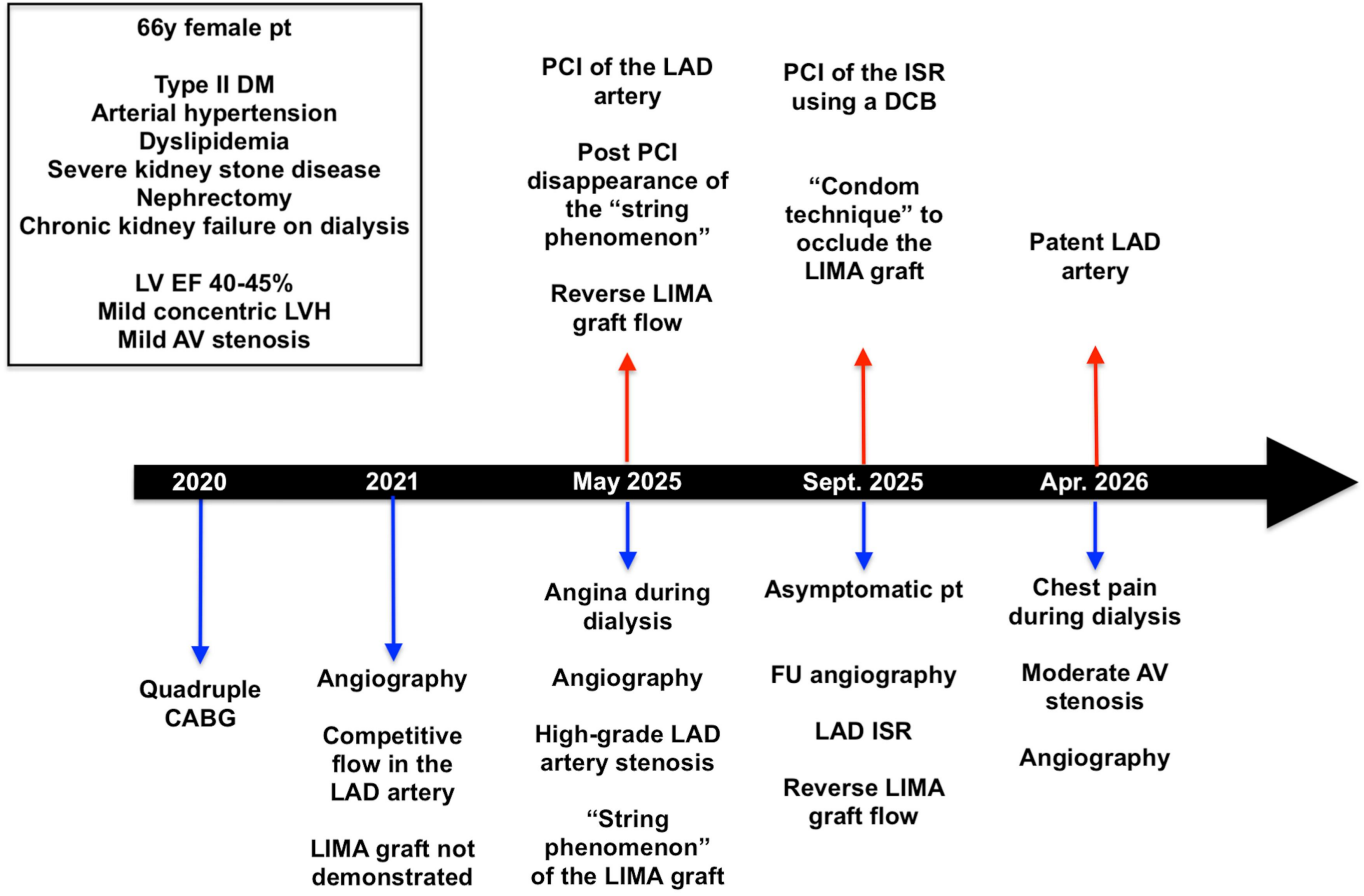
A timeline summarizing the main events of this case report.

A 66-year-old woman patient, with a history of insulin-dependent type II diabetes mellitus, arterial hypertension, dyslipidemia, nephrectomy for severe kidney stone disease, chronic kidney failure on dialysis, and quadruple CABG (LIMA to the LAD artery and SVG to the second diagonal, obtuse marginal, and right posterior descending arteries) performed in 2020, underwent invasive angiography because of angina during a hemodialysis session. An ECG showed a sinus rhythm and signs of complete left bundle branch block, which was a pre-existing finding, without any of the Smith-modified Sgarbossa criteria for myocardial infarction. An echocardiography was remarkable for a left ventricular ejection fraction of 40%−45%, concentric mild left ventricular hypertrophy, and mild aortic valve stenosis. An angiography showed patent SVGs, a high-grade stenosis in the proximal-to-mid segment of a wrap-around LAD artery, and a diffuse narrowing of the LIMA graft (“string phenomenon”) (Figure 1A, Supplementary Videos S1, S2). The LIMA graft was not injected because its ostium could n't be spotted. An angiogram performed in 2021 revealed competitive flow in the mid/distal LAD artery after injection into the left coronary artery system; however, the LIMA graft was not injected (Supplementary Video S3). We performed a successful intravascular ultrasound (IVUS; 20 MHz Eagle Eye catheter; Philips)-guided PCI of the LAD artery with the implantation of a 2.75 × 38 mm drug-eluting stent (DES) (Figure 1B). Reverse flow through an unobstructed LIMA graft was observed immediately after the PCI (Supplementary Videos S4, S5). Subsequent selective engagement of the LIMA graft showed competitive flow (Supplementary Video S6). A duplex ultrasound excluded a left subclavian artery stenosis proximal to the LIMA ostium, which could have caused the reverse LIMA graft flow. In order to evaluate the status of the LIMA graft, we performed angiography three and a half months later while the patient was asymptomatic, which revealed ISR and retrograde flow opacification of the LIMA graft, the selective injection of which showed competitive flow (Figures 1C,D, Supplementary Videos S7–S9). The ISR lesion comprised echolucent neointimal tissue at the site of a well-expanded stent (Figure 1C; embedded panel) and was successfully treated by angioplasty using a 3.0 × 30 mm paclitaxel drug-coated balloon (DCB) (Figures 1E,F, Supplementary Video S10). Because the ISR might have been an adverse sequela of the competitive flow from an anatomically patent but dysfunctional LIMA graft, we used the “condom technique” to occlude it. First, we determined the diameter of the LIMA graft close to the distal anastomosis using IVUS. A cut remnant of the distal part of a 2.25 mm angioplasty balloon mounted on a 2.25 mm stent was then delivered proximal to the distal LIMA graft anastomosis (Figure 1G), followed by stent deployment (Figure 1H), achieving the occlusion of the graft (Figure 1I, Videos S11, S12). This was achieved on the second attempt to apply the technique because the first attempt was complicated by a dislodgment of the remnant balloon from the assembly and its migration into the distal LAD artery. The dislodged remnant balloon was deliberately wedged in the distal part of the wrap-around LAD artery, causing its occlusion without any adverse sequelae. The patient was discharged home in good condition on guideline-recommended medical therapy, including dual antiplatelet therapy with aspirin and clopidogrel for 3 months due to a high bleeding risk emanating from end-stage kidney disease and a hemoglobin concentration <11.0 g/dL. The woman remained free of angina or any cardiovascular events at a 6-month follow-up. Seven months after the latest PCI, the patient was referred for invasive angiography because of chest pain during a hemodialysis session without specific signs of ischemia on the electrocardiogram. This time, the stenosis of the aortic valve progressed to a moderate degree. An angiography showed patent SVGs and preserved PCI results on the LAD artery (Supplementary Video S13). The patient was discharged home with instructions for regular cardiology follow-up. A timeline summarizing the main events of this case report is presented in Figure 2.

## Discussion

Competitive flow between a LIMA graft and a LAD artery containing a non-flow-limiting stenosis or supplying an infarcted myocardial territory may lead to diffuse graft narrowing (“string phenomenon”), usually identified within the first year post-CABG, and graft failure in the majority of patients (2–5). Such a scenario implies that these vessels have similar conductance, allowing for an equilibrium of residual flow between them, and is related to a significant reduction in graft flow, mainly during diastole, thereby producing a low-flow state. The latter causes the “string phenomenon,” which may reverse when the degree of stenosis within the grafted LAD artery or the corresponding myocardial oxygen demand increases. However, over time, endothelial dysfunction ensues, leading to intimal hyperplasia and graft atherosclerosis, and ultimately, graft failure (2, 7, 9). Although the negative impact of competitive flow on LIMA graft patency seems to be gradual and mild, the less severe the proximal coronary artery stenosis is, the greater will be the competitive flow, making it more likely for the LIMA graft to occlude (5, 6). Also, at least down to a 40% diameter stenosis, there is no specific degree of proximal coronary artery stenosis severity below which competitive flow abruptly increases and LIMA graft patency abruptly decreases, suggesting that other, yet unknown factors potentially play a role in LIMA graft occlusion in such cases.

In the case of our patient, an angiogram was performed prior to CABG because of an inducible ischemia of the apex, apical septum, and apical inferior wall observed on a dobutamine stress echocardiogram, indicating a high likelihood of significant, flow-limiting (≥50%) lumen diameter stenosis of the LAD artery. Indeed, an angiographically high-grade (≥70%) LAD artery stenosis was observed (Supplementary Figure S1). However, given the moderate accuracy of dobutamine stress echocardiography in detecting hemodynamically significant coronary artery disease, as indicated by fractional flow reserve, the possibility of a false-positive result cannot be excluded (13). Nevertheless, because a 40%–90% lumen diameter stenosis may produce competitive flow, the observation of such a flow pattern 1 year after CABG in the presence of an angiographically high-grade stenosis was anticipated (2). A LIMA graft, by virtue of its functional vascular smooth muscle cells, retains the ability to adapt its diameter in response to myocardial oxygen demand (6, 7). Therefore, with the progression of native coronary artery stenosis, the diameter of the LIMA graft increases, leading to increased flow reserve and decreased flow velocity. In contrast, when the severity of native coronary artery stenosis decreases, which can sometimes occur after LIMA grafting, the competitive flow increases. This, in combination with a reduced need for blood supply through the LIMA graft, leads to graft constriction, thereby increasing the risk of graft occlusion over time (1, 6, 14). In our patient, the grafted diagonal artery was occluded proximal to an SVG anastomosis, thereby preventing retrograde flow and, consequently, competitive flow in the LAD artery, which could help increase the already present competitive flow, thus increasing the risk of LIMA graft failure (3, 5). Nor was there an unligated LIMA branch that could account for the “string phenomenon” secondary to a steal mechanism (8). Accordingly, one could infer that increased competitive flow related to an initially hemodynamically significant LAD artery stenosis, which might have regressed following LIMA grafting, or a hemodynamically non-significant LAD artery stenosis, led to the “string phenomenon” at some time point after the first year post-CABG. Given that the wrap-around LAD artery supplied a viable myocardium and the stenosis of the LAD artery was angiographically high grade, the LIMA graft should n't have displayed the “string phenomenon” if it had been functional. In other words, if the “string phenomenon” were an initial adaptive response to a low-flow state, one would expect it to revert, restoring the patency of the LIMA graft once severe stenosis in the LAD artery developed. This would cause a fall in the distal pressure, thereby creating a need for increased flow through the LIMA graft in order to match the physiologic demand of the corresponding myocardial territory (2, 7, 15). Furthermore, the failure of the LIMA graft to recruit functional patency suggests that the “string phenomenon” might have developed a considerable time ago, allowing permanent anatomical changes to the wall of the LIMA graft, such as intimal hyperplasia and atherosclerosis (2). Indeed, our patient showed atherosclerosis of the LIMA graft, as demonstrated by IVUS (Supplementary Video S14).

Upon revascularization of the LAD artery, the “string phenomenon” vanished, and a dilated LIMA graft exhibited reverse flow, a clear indication of severe competitive flow and the functional occlusion of the graft. Although the patient's coronary artery disease risk factors have certainly played a role in the development of stent failure, the persisting competitive flow was viewed as another modifiable contributing factor causing diminished flow in the proximal LAD artery and therefore low and oscillatory wall shear stress, in turn, modulating the proliferative and migratory responses of the vascular smooth muscle cells, thereby facilitating neointimal hyperplasia (10–12). Given the asymptomatic status of our patient and the fact that the expected progression of the stenosis at the site of the ISR would, over time, create a demand for increased flow through the LIMA, which could help recruit the dysfunctional graft, it would be better to advocate a watchful waiting strategy. In-stent restenosis, however, is a complex disease entity manifesting as an acute coronary syndrome in up to 20% of patients, which portends an increased risk of adverse cardiovascular events (16). Furthermore, patients undergoing hemodialysis are particularly susceptible to myocardial ischemia because of a reduced coronary flow reserve caused by coronary microvascular dysfunction and diffuse epicardial coronary artery atheromatosis, a reduced ischemic threshold owing to reduced peripheral arterial compliance and left ventricular hypertrophy, and defective vasoregulation, which increases the risk of intradialytic hypotension and reduced cardiac output (17). Choosing not to address the ISR, thereby leaving a potentially flow-limiting stenosis untreated in the presence of a dysfunctional LIMA graft—whose time of potential recruitment is unknown, if it occurs at all—would mean that our patient would face an even higher risk of myocardial ischemia every time she undergoes dialysis. Furthermore, if the LIMA graft were to become functional again, our patient would face an ongoing risk for acute coronary syndrome throughout the period prior to the graft's recruitment. Consequently, addressing the ISR and eliminating the source of competitive flow by deliberately occluding the dysfunctional LIMA graft was deemed a more prudent course of action. Indeed, the preserved patency of the LAD artery seven months after DCB angioplasty for the ISR and occlusion of the LIMA graft demonstrates how harmful the persistent competitive flow was in relation to the ISR risk. Although current guidelines advocate repeat DES implantation as first-line therapy for the first occurrence of DES ISR, given the biological nature of the ISR in our patient and optimal lesion preparation, we chose to address the lesion using a DCB, a strategy supported by the DCB Academic Research Consortium (18). Considering the fact that the ISR occurred only three and a half months after the index PCI, the neointimal tissue must have comprised proteoglycan-rich fibrous tissue with relatively few smooth muscle cells (19). Indeed, we chose to use a paclitaxel DCB because the trapping and binding of paclitaxel is facilitated by the proteoglycan-rich extracellular matrix found in the neointimal tissue, something that very likely contributed to an enhanced antiproliferative action of the drug and the good outcome demonstrated 7 months later (18). Because there is no “class effect” in DCB angioplasty, should our patient develop recurrent ISR, we could treat her with a different brand of DCB or with repeat DES implantation guided by intravascular imaging, provided that the lesion has been adequately prepared.

Intentional occlusion of vascular grafts by deployment of coils or vascular plugs to nullify the potentially deleterious effects of competitive flow after a successful PCI of the corresponding native coronary artery has been proposed as a safe, highly successful, and effective procedure (20). The lack of experience with the abovementioned techniques led us sacrifice the LIMA graft using the “condom technique.” This does not require any specific equipment and involves trapping the proximal part of a cut remnant of the distal part of an angioplasty balloon between the deployed stent and the graft's wall, thereby transforming the lumen into a dead end (21, 22). The successful application of this technique requires coaxial guide catheter engagement at the ostium of the target graft and unimpeded navigation of the remnant balloon–stent assembly to the desired position (22). The inability to retrieve the remnant balloon component of the assembly in cases where the deliverability of the assembly is constrained represents a potential drawback of the “condom technique.” Accordingly, if the guide catheter is misaligned with the ostium of the graft, or the ostium is angulated or narrowed, inadvertent pushing of the assembly may result in loss of the guide catheter and guidewire position with subsequent embolization of the remnant balloon in the systemic circulation. Similarly, the remnant balloon may become stuck in an angulated part of the graft, where, depending on the specific circumstances of the blood flow, it may migrate either proximally or distally and embolize at a later time point either in the systemic circulation or in the corresponding native coronary artery (23). A stuck remnant balloon may also thrombose, thereby becoming a nidus of thromboemboli in the distal native coronary artery circulation, resulting in an acute coronary syndrome. In our patient, we chose to deploy a guideliner catheter in the distal part of the LIMA graft in order to overcome its tortuous proximal segment, which could pose difficulties in smoothly navigating the remnant balloon–stent assembly to the desired position proximal to the LIMA graft anastomosis. Because of single arterial access, we relied on two hemostatic clips which were used to ligate the LIMA branches and were located close to the distal LIMA graft anastomosis, to position the remnant balloon–stent assembly, so that the marker at the distal end of the remnant balloon would be located at the site of the clips. After having deployed the remnant balloon–stent assembly to the desired position using the 30-degree right anterior oblique projection in which the distal LIMA graft anastomosis was clearly visible, we attempted to confirm its position by gently injecting 1 mL of contrast medium. This caused dislodgment of the remnant balloon from the assembly and migration into the distal LAD artery. The dislodged remnant balloon was then deliberately wedged in the distal part of the wrap-around LAD artery, causing its occlusion without any adverse sequelae. Another remnant balloon–stent assembly was then constructed and deployed successfully, achieving the occlusion of the graft. Unlike in our patient, choosing to apply this technique using double arterial access might be safer because it allows angiography of the revascularized native coronary artery, during which retrograde filling of the target graft helps assess the position of the remnant balloon–stent assembly.

## Conclusion

We present the case of a patient with unstable angina and a failed LIMA graft to the LAD artery, exhibiting the “string phenomenon” because of competitive flow, necessitating a PCI of a high-grade stenosis in the LAD artery. This case highlights the complex, flow-dependent interactions between a LIMA graft and a grafted LAD artery. The “string phenomenon” was no longer present immediately after the revascularization of the LAD artery; it did not recur during follow-up, and the LIMA graft maintained a reverse flow, indicating severe and persistent competitive flow, which was implicated in the development of ISR. In such patients, deliberate occlusion of the LIMA graft may help reduce the risk of future episodes of stent failure. Indeed, elimination of the persistent competitive flow by occlusion of the dysfunctional LIMA graft had a favorable impact on the patient's outcome, as testified by the fact that seven months after the DCB angioplasty for the ISR, the LAD artery was shown to be patent. As presented herein, choosing to sacrifice the graft using the “condom technique” is feasible, safe, and effective, provided all steps of the technique are thoroughly reviewed and optimally performed.

## Data Availability

The original contributions presented in this study are included in the article/Supplementary Material. Further inquiries can be directed to the corresponding authors.

## Supplementary material

Supplementary Figure S1 Right anterior oblique cranial angiographic view prior to bypass graft surgery demonstrating a high-grade (≥70%) stenosis (arrow) in the proximal-to-mid segment of a wrap-around left anterior descending artery.

Supplementary Video S1 Angiography in the right anterior oblique cranial projection depicting a high-grade stenosis in the proximal-to-mid segment of a wrap-around LAD artery. Note the “string phenomenon” of the LIMA graft.

Supplementary Video S2 Angiography in the left lateral 90° projection depicting a high-grade stenosis in the LAD artery. Note the “string phenomenon” of the LIMA graft.

Supplementary Video S3 Angiography of the left coronary artery system in the right anterior oblique cranial projection performed one year after LIMA-to-LAD artery grafting, depicting competitive flow in the mid/distal LAD artery.

Supplementary Video S4 Angiography in the right anterior oblique cranial projection depicting a good result after stent angioplasty of the LAD artery. Note the reverse flow through a dilated LIMA graft.

Supplementary Video S5 Angiography in the right anterior oblique caudal projection depicting a good result after stent angioplasty of the LAD artery. Note the reverse flow through a dilated LIMA graft.

Supplementary Video S6 Angiography of the LIMA graft depicting competitive flow.

Supplementary Video S7 Angiography in the right anterior oblique cranial projection depicting in-stent restenosis of the LAD artery. Note the reverse flow through a dilated LIMA graft.

Supplementary Video S8 Angiography in the right anterior oblique caudal projection depicting in-stent restenosis of the LAD artery. Note the reverse flow through a dilated LIMA graft.

Supplementary Video S9 Follow-up angiography of the LIMA graft depicting competitive flow.

Supplementary Video S10 Angiography in the right anterior oblique cranial projection depicting a good result after drug-coated balloon angioplasty of the in-stent restenosis of the LAD artery. Note the reverse flow through a dilated LIMA graft.

Supplementary Video S11 Angiography in the right anterior oblique cranial projection depicting occlusion of the LIMA graft following the application of the “condom technique”. Note that the distal part of the wrap-around LAD artery is occluded from the remnant balloon which dislodged from the assembly during the first attempt to perform the “condom technique”.

Supplementary Video S12 Angiography in the right anterior oblique caudal projection depicting occlusion of the LIMA graft following the application of the “condom technique”. Note that the distal part of the wrap-around LAD artery is occluded from the remnant balloon which dislodged from the assembly during the first attempt to perform the “condom technique”.

Supplementary Video S13 Angiography in the right anterior oblique cranial projection seven months after the angioplasty for in-stent restenosis of the LAD artery and deliberate occlusion of the LIMA graft depicting a patent LAD artery stent.

Supplementary Video S14 Intravascular ultrasound run of the left internal mammary artery graft.
