# Supplementary material for: Case Report: Navigating the flow-dependent interaction between the left internal mammary artery graft and the left anterior descending artery: when deliberate graft occlusion comes into play
Source: Front Cardiovasc Med. 2026 Aug 4;13:1865177. doi: 10.3389/fcvm.2026.1865177 (PMC13481196; doi:10.3389/fcvm.2026.1865177)
Supplement: Supplementary file 1 [file Table1.docx]

**Supplementary material online**

**Figure S1**

Right anterior oblique cranial angiographic view prior to bypass graft surgery demonstrating a high-grade (≥70%) stenosis (arrow) in the proximal-to-mid segment of a wrap-around left anterior descending artery.

**Video S1**. Angiography in the right anterior oblique cranial projection depicting a high-grade stenosis in the proximal-to-mid segment of a wrap-around LAD artery. Note the “string phenomenon” of the LIMA graft.

**Video S2**. Angiography in the left lateral 90° projection depicting a high-grade stenosis in the LAD artery. Note the “string phenomenon” of the LIMA graft.

**Video S3**. Angiography of the left coronary artery system in the right anterior oblique cranial projection performed one year after LIMA-to-LAD artery grafting, depicting competitive flow in the mid/distal LAD artery.

**Video S4**. Angiography in the right anterior oblique cranial projection depicting a good result after stent angioplasty of the LAD artery. Note the reverse flow through a dilated LIMA graft.

**Video S5**. Angiography in the right anterior oblique caudal projection depicting a good result after stent angioplasty of the LAD artery. Note the reverse flow through a dilated LIMA graft.

**Video S6**. Angiography of the LIMA graft depicting competitive flow.

**Video S7**. Angiography in the right anterior oblique cranial projection depicting in-stent restenosis of the LAD artery. Note the reverse flow through a dilated LIMA graft.

**Video S8**. Angiography in the right anterior oblique caudal projection depicting in-stent restenosis of the LAD artery. Note the reverse flow through a dilated LIMA graft.

**Video S9**. Follow-up angiography of the LIMA graft depicting competitive flow.

**Video S10**. Angiography in the right anterior oblique cranial projection depicting a good result after drug-coated balloon angioplasty of the in-stent restenosis of the LAD artery. Note the reverse flow through a dilated LIMA graft.

**Video S11**. Angiography in the right anterior oblique cranial projection depicting occlusion of the LIMA graft following the application of the “condom technique”. Note that the distal part of the wrap-around LAD artery is occluded from the remnant balloon which dislodged from the assembly during the first attempt to perform the “condom technique”.

**Video S12**. Angiography in the right anterior oblique caudal projection depicting occlusion of the LIMA graft following the application of the “condom technique”. Note that the distal part of the wrap-around LAD artery is occluded from the remnant balloon which dislodged from the assembly during the first attempt to perform the “condom technique”.

**Video S13.** Angiography in the right anterior oblique cranial projection seven months after the angioplasty for in-stent restenosis of the LAD artery and deliberate occlusion of the LIMA graft depicting a patent LAD artery stent.

**Video S14.** Intravascular ultrasound run of the left internal mammary artery graft
